# Hypothesis: solid tumours behave as systemic metabolic dictators

**DOI:** 10.1111/jcmm.12794

**Published:** 2016-02-04

**Authors:** Yang‐Ming Lee, Wei‐Chun Chang, Wen‐Lung Ma

**Affiliations:** ^1^ Sex Hormone Research CenterGraduate Institution of Clinical Medical ScienceChina Medical UniversityTaichungTaiwan; ^2^ Department of Endocrinology and MetabolismChanghua Christian HospitalChanghuaTaiwan; ^3^ Sex Hormone Research CenterDepartment of Gynecology and ObstetricChina Medical University HospitalTaichungTaiwan

**Keywords:** endocrine organ–like tumour, cancer macroenvironment, paraneoplastic syndrome, cancer cachexia

## Abstract

Current knowledge regarding mechanisms of carcinogenesis in human beings centres around the accumulation of genetic instability, amplified cellular signalling, disturbed cellular energy metabolism and microenvironmental regulation governed by complicated cell–cell interactions. In this article, we provide an alternative view of cancer biology. We propose that cancer behaves as a systemic dictator that interacts with tissues throughout the body to control their metabolism and eventually homeostasis. The mechanism of development of this endocrine organ–like tumour (EOLT) tissue might be the driving force for cancer progression. Here, we review the literature that led to the development of this hypothesis. The EOLT phenotype can be defined as a tumour that alters systemic homeostasis. The literature indicates that the EOLT phenotype is present throughout cancer progression. The feedback mechanism that governs the interaction between tumours and various organs is unknown. We believe that investigating the mechanism of EOLT development may advance the current knowledge of regulation within the tumour macroenvironment and consequently lead to new diagnostic methods and therapy.

## Introduction

Cancer is one of the most complicated diseases and its mechanism of development is still largely unknown. Decades of investigation have revealed the following cancer hallmarks, such as sustained proliferative signalling, evasion of growth suppressors, resistance to cell death, replicative immortality, angiogenesis induction and proneness to invade and metastasize, promote inflammation, deregulate cellular energy, avoid immune destruction and induce genome instability and mutation 1. Metabolic reprogramming has emerged as another hallmark feature, but the underlying mechanism remains elusive. The most distinctive metabolic differences from normal tissues are increased aerobic glycolysis 2, elevated glutaminolytic flux 3, 4 and enhanced amino acid and lipid metabolism 5. Cancer cells sustain high metabolic turnover rates to ensure sufficient biomass synthesis. A large amount of energy is required to support this process. However, current knowledge regarding tumour homeostasis mainly focuses on the tumour itself, but does not consider effects on the whole body. Here, we discuss tumour biology from an energy homeostasis point of view and discuss the insufficiency of current theories. In this study, we aimed to provide an updated review of important aspects of cancer biology and to establish the systemic as well as central effects of the tumour as an endocrine‐like organ. Further investigations on this hypothesis would help early diagnosis, prevention and disease intervention of cancer.

### The energy demand and supply domains of solid tumours

In the 1920s, Otto Warburg first observed that glycolysis and lactate production are increased in cancer cells regardless of oxygen availability, a phenomenon known as ‘the Warburg effect’ 2. In contrast to normal cells, which produce energy primarily by mitochondrial respiration, cancer cells synthesize adenosine triphosphate (ATP) *via* aerobic fermentation 2. One molecule of glucose generates only two ATP molecules *via* aerobic glycolysis rather than the 36 ATP molecules that are generated through mitochondrial oxidative phosphorylation 6.

Elevated glucose uptake and utilization is a trait of many human cancers, widely used to identify primary and metastatic lesions by positron emission tomography with 18F‐fluorodeoxyglucose 7, 8, 9. It remains largely unclear why tumour cells utilize a less efficient method of energy metabolism. One explanation is that nutrient uptake and metabolic shift accelerate the synthesis of biological building blocks (*e.g*. amino acids, nucleic acids and lipids) needed for rapid cancer cell division 10, 11, 12. Another explanation is that ATP is generated faster from aerobic glycolysis than from mitochondrial respiration since cancer cells have a continuous supply of glucose 10, which facilitates rapid cancer cell growth. One possible advantage of aerobic glycolysis is that it generates less reactive oxidative species (ROS) than the mitochondrial respiratory chain 13, 14, 15. However, energy utilization is inefficient, which can harm cancer cell metabolism even while promoting cancer progression. The mechanism by which cancer cells prevent the accumulation of aerobic glycolysis by‐products is unknown.

Glutamine also provides energetic fuel for certain cancer cells 3, 4. Many tumours rely on glutaminolysis for energy production and metabolic adaptation 3, 4, 11. The products of glutaminolysis are essential to replenish the intermediates of the tricarboxylic acid (TCA) cycle, which are utilized for the synthesis of lipid, cholesterol, amino acids and other metabolites 5. Additionally, NADH and FADH2 from the TCA cycle supply electrons to the mitochondrial respiratory chain to generate ATP. Cancer cells can consume both glucose and glutamine to generate NADPH. This allows tumours to fuel biomass synthesis and restores the reducing power of both glutathione and thioredoxin, which scavenge ROS generated during rapid proliferation 14, 16.

### Genetic alterations in tumour homeostasis

Tumour cells are more resistant than normal cells to the toxic, acidic environment created by aerobic glycolysis 10, placing metabolism in the service of lengthening survival and increasing proliferation under stress conditions. Many changes to Warburg phenomenon‐related molecular pathways, either genetic mutations or alterations of the tumour microenvironment, are involved. Interestingly, those genetic changes related to metabolic shift are pro‐tumourigenic 17. Alterations of the most crucial transcription factors, including p53 18, c‐myc 19 and hypoxia‐inducible factor 1 (HIF1) 20, promote metabolic transformation in cancer 21. For example, mutations in the tumour suppressor gene, p53, inhibit the expression of the glucose transporters GLUT1, GLUT3 and GLUT4 and suppress glycolysis *via* inhibition of the phosphatidylinositol‐3‐kinase (PI3K) pathway 22, 23. Therefore, inactivation of p53 increases glucose uptake and utilization 24. On the other hand, the proto‐oncogene c‐Myc is constitutively expressed in tumour cells and HIFs are activated in response to hypoxia 25. Both HIF1 and c‐MYC expression enhance the glycolytic pathway through elevation of GLUT1, GLUT4, pyruvate kinase (PK) and lactate dehydrogenase A (LDH‐A) 26. Furthermore, c‐MYC stimulates glutamine uptake and metabolism 27. p53 also induces expression of the mitochondrial glutaminase encoding gene, thereby increasing energy production from glutaminolysis 28.

### Altered kinase activity and mitochondrial pathways lead to changed glucose utilization

Apart from the activation of transcription factors, altered activity of key metabolic enzymes, such as AMP‐activated protein kinase (AMPK) 29 and PK isoform M2 (PKM2) 30, regulated by growth factors and transcription factors, are also landmarks of cancer development. AMPK is an important response to glucose starvation, and its activity is controlled by cellular levels of adenylates (*e.g*. ATP, ADP or AMP) 31. In response to low ATP/AMP, AMPK can activate the expression of genes for survival and metabolic adaption through histone H2B phosphorylation on serine 36 32. Hence, AMPK can be a nutrient/energy sensor whose activation can influence metabolism and energy balance under metabolic stress by altering various pathways, including up‐regulation of GLUT 31, mitophagy 33, 34, fatty acid oxidation (FAO) 35 and even appetite 31.

Pyruvate kinase, which catalyses the rate‐limiting step of glycolysis, is also important 36. The constitutively active form, PKM1, is found in most adult tissues, whereas the alternate form, pyruvate kinase isoform 2 (PKM2), is highly expressed in embryonic and proliferating tissues that depend on glycolysis 37. In tumour cells, mTOR up‐regulates PKM2 to promote aerobic glycolysis, which in turn (through HIF1 and c‐Myc) enhances cell survival in different oxygen and nutrient gradient microenvironments 36, 38.

Mitochondrial genetic changes are another key feature of cancer metabolic transformation. Mitochondrial dysfunction was thought to facilitate glycolysis as described by Warburg 2; however, studies indicate that mitochondrial DNA mutations do not inactivate mitochondrial energy metabolism but rather change mitochondrial bioenergetic and biosynthetic status 39. In this scenario, mitochondria are not merely the powerhouse of the cells but also the factories providing critical molecules for cellular biosynthesis, growth and proliferation. Mutations in the mitochondrial genes for the TCA cycle 39, including succinate dehydrogenase (SDH) and fumarate hydratase (FH), are well recognized in cancer cells. Germline mutations of the different SDH subunits have been found in paraganglioma and pheochromocytoma 40. Mutations in FH are responsible for the development of leiomyoma, leiomyosarcoma and renal clear cell carcinoma 41, 42. Mutations of SDH and FH increase levels of fumarate and succinate, which curb α‐ketoglutarate‐dependent prolyl hydroxylases, thereby leading to stabilization of HIF1α 43. The stabilized HIF1α enhances glycolysis and triggers tumourigenesis 41, 44. Thus, mutations in metabolic enzymes not only alter metabolism but also contribute to carcinogenesis.

### Alternative sources of cellular energy in tumour homeostasis

Another remarkable change in cancer metabolism is the increase in lipid metabolism. Lipids are essential building blocks of organelle membranes and the fuel of cancer cells. Cancer cells frequently up‐regulate *de novo* fatty acid synthesis to satisfy their need for lipids 45, 46. Fatty acids are a rich energy resource that can yield far more ATP than glucose. Recently, ATP derived from FAO was shown to inhibit anoikis 47, a type of cell death triggered by loss of matrix attachment 48. Fatty acid oxidation also provides NADPH to protect against ROS 49. Thus, FAO can improve cancer survival by increasing energy production and the supply of precursors and by quenching oxidative stress.

Cholesterol is an integral component of biological membranes as it regulates the fluidity of the lipid bilayer and determines membrane organization and properties 50. Cholesterol contributes to the conformation of lipid rafts that coordinate the activation of several signalling pathways 51. The mevalonate (MVA) pathway is a core biosynthesis pathway, critical for the generation of cholesterol and other fundamental end‐products that are necessary for cell growth and proliferation, such as geranylgeranyl pyrophosphate and farnesyl pyrophosphate. These isoprenylated molecules are required for some cancer‐relevant signalling cascades (such as Akt and PI3K) and signalling molecules such as small GTPase activating proteins 52. Intriguingly, evidence shows that hydroxymethylglutaryl coenzyme A reductase, the rate‐limiting enzyme of MVA pathway, is a candidate metabolic oncogene and plays a role to promote cell transformation 53. Besides increased biosynthesis, intracellular cholesterol accumulation can be mediated by low‐density lipoprotein receptor. A recent study reveals that low‐density lipoprotein receptors are highly expressed in pancreatic tumour cells 54. This allows the tumour to meet its excessive cholesterol demand during carcinogenesis. However, how solid tumours make cholesterol remains elusive.

### The role of the tumour microenvironment in regulating tumour energy homeostasis

As described above, both intrinsic and extrinsic factors profoundly affect metabolic phenotype. During tumourigenesis, cancer cells encounter a hostile environment characterized by hypoxia, acidity and nutrition deprivation 55. When hypoxia occurs, HIFs sense the microenvironmental change in oxygen concentration and coordinate the metabolic switch away from mitochondrial respiration towards glycolysis 20, 56, 57. However, cancer cells can utilize glycolysis without being exposed to hypoxic conditions. For example, leukaemic cells and lung tumours have high rates of glycolysis even in high oxygen environments 37. In addition, hypoxia, combined with deficient vessel perfusion and high glucose consumption, contributes to the acidification of the extracellular environment. This effect is mainly attributed to increased extrusion of H^+^ and lactate from tumour and stromal cells 58 and is shown to alter the metabolism and function of immune cells by dampening T‐cell receptor activation, cytotoxic secretion and cytokine activity of T lymphocytes. Collectively, these effects cause a weakening of the immune response to tumour cells 59, 60. These results underscore the functional importance of tumour energy homeostasis in a complicated cell–cell communication and paracrine network.

In 1889, Stephen Paget first described the ‘seed and soil’ hypothesis, which states that a cancer cell seeds or grows in the host tissues as if in fertile soil 61. In fact, solid tumour cells are composed of stromal tissues, including fibroblasts, adipocytes, resident epithelial cells, vessel cells and infiltrating immune cells 62. Studies indicate that cancer‐associated fibroblasts are the most common cells within the tumour microenvironment 62. Cancer‐associated fibroblasts facilitate tumour cell growth and cancer progression. Likewise, cancer cells produce growth factors that activate and recruit cancer‐associated fibroblasts 62. Cancer‐associated fibroblasts spur tumour growth by supplying not only cytokines and growth factors but also nutrients 63. Cancer‐associated fibroblasts produce energy‐rich metabolites, such as L‐lactate, ketone, free fatty acids and glutamine, *via* catabolism (through autophagy, mitophagy and aerobic glycolysis). These metabolites are transferred to the mitochondrial respiratory machinery of adjacent epithelial cancer cells 64. This is called the ‘reverse Warburg effect’ because glycolysis increases in stromal cells rather than the tumour cells 64. Monocarboxylate transporter 4 (MCT4), the predominant exporter of lactate and other monocarboxylates (such as ketone bodies) out of the cell, is up‐regulated in stromal cells and is a marker of glycolysis 65. In contrast, MCT1, a main transporter for the uptake of lactate and ketone bodies, is up‐regulated in cancer cells and is a marker of oxidative phosphorylation 65. Monocarboxylate transporter 4 is regulated through hypoxia and HIF1 66, while MCT1 is regulated by Myc 67. Therefore, energy transfer between cancer cells and host tissues is necessary to maintain metabolic homeostasis during tumour progression 68. More importantly, this highlights the significance of cell–cell interactions between solid tumour and the host needed by cancer cells to meet their energy demands.

### Current knowledge of the unmet metabolic demands of tumour cells

Cancer cells require energy to synthesize the building blocks needed for cell proliferation. Acquiring nutrients from adjacent stromal cells or increasing glucose uptake may be insufficient to satisfy the demand imposed by tumour progression. Consequently, obtaining energy from distant organs may be crucial to maintenance of tumour energy homeostasis. To force host tissue to release energy, cancer cells secrete many factors that defeat physiological hormone regulation, thereby taking control of energy balance. For example, tumours might increase insulin resistance through the production of inflammatory cytokines [*e.g*. tumour necrosis factor alpha (TNF‐α)] that cause down‐regulation of GLUT4 69. Moreover, lactate could be transported from cancer cells to liver cells, which then use it as a gluconeogenesis substrate. Hence, both strategies feed tumour cells by causing hyperglycaemia.

Diabetes and cancer are affected by genetic and similar modifiable environmental risk factors, including obesity and low physical activity. Epidemiological studies show that diabetic (predominately type 2) patients (compared with non‐diabetic patients) have a higher incidence and mortality rate from cancers, including liver, endometrium, pancreas, stomach, kidney, bladder and breast cancers 70. However, the mechanisms underlying the association of diabetes with higher cancer risk are unclear. Hyperglycaemia‐induced increase in glucose supply to fuel tumour growth may explain diabetes‐associated risk. Alternatively, tumours might induce hyperglycaemia by enhancing insulin resistance and hepatic gluconeogenesis, thus providing extra glucose required for tumour growth.

Diabetes‐induced hyperglycaemia, hyperinsulinemia and inflammation can potentially promote cancer development. Studies performed *in vitro* showed that activation of the insulin receptor (IR) or insulin‐like growth factor‐1 receptor augments proliferation and inhibits apoptosis of cancer cells. Moreover, several metabolism‐related factors, such as obesity, low physical activity, Western diets, anti‐hyperglycaemia agents and smoking, have been identified as risk factors for cancer. The connection between obesity and cancer progression is believed to disrupt signal transduction and alter levels of adipocyte‐derived factors, such as adipokines, leptin, plasminogen activation inhibitor‐1 and pro‐inflammatory cytokines 71. Consequently, it is believed that anti‐hyperglycaemia agents might promote cancer progression; however, different anti‐hyperglycaemia agents produce different outcome among cancer patients. Studies have shown that metformin reduces cancer risks 72, while sulfonylureas and insulin therapy increase cancer risk 73. These conflicting results may be the result of reduced insulin resistance caused by metformin and increased hyperinsulinemia caused by sulfonylureas and exogenous insulin therapy. Therefore, it is likely that a regulatory circuit connects the tumour and its microenvironment with the larger environment of the organism.

## Materials and methods

We conducted a PubMed literature search for studies about cancer metabolism using the key phrases ‘cancer hallmarks’, ‘cancer metabolism’, ‘Warburg effect’, ‘metabolic reprogramming’, ‘cancer microenvironment’, ‘cancer macroenvironment’, ‘cancer and diabetes’, ‘cancer cachexia’, ‘cancer cachexia and Cori cycle’, ‘paraneoplastic syndrome’, ‘cancer and cholesterol’ and ‘cancer and microbiota’.

Our search strategy was to match the titles with our search first and then to evaluate the linkage or relevance to our hypothesis. For instance, the search containing the key phrase ‘cancer hallmarks’ found 2123 articles and that containing the key phrase ‘cancer metabolism’ found 397,575 articles. However, only two or three studies in the respective searches met our inclusion criteria and so on.

## Hypothesis

We hypothesized that solid tumours behave as systemic metabolic dictators and control whole body homeostasis in an endocrine organ–like manner. We believe that solid tumours and peripheral organs interact with each other in a regulatory feedback and continually evolving manner during tumour development.

## Results

### Supporting evidence

#### Findings in early‐stage disease

Supportive evidence for our hypothesis includes the well‐established observation that some cancers can cause paraneoplastic syndromes (*i.e*. a group of clinical disorders due to functional peptide and hormone produced by tumours or immunological cross‐reactivity between tumour and normal host tissues rather than tumour development itself) 74. For example, paraneoplastic Cushing syndrome arises from tumour secretion of adrenocorticotropic hormone or corticotropin‐releasing factor 75, 76. Approximately 50–60% of these cases are small‐cell lung cancers and bronchial carcinoid tumours 76, and most patients often present with symptoms of Cushing syndrome before cancer is diagnosed 76. Furthermore, around 85% of patients with pancreatic cancer develop diabetes or hyperglycaemia, which often present as early as 2–3 years before pancreatic cancer is diagnosed 77. Diabetes (hyperglycaemia) and weight loss, which manifest several months before the onset of cachexia, are paraneoplastic phenomena induced by pancreatic cancer 78. Adrenomedullin mediates β‐cell dysfunction in pancreatic cancer‐induced diabetes 79. Taken together, these findings seem to suggest that solid tumours are endocrine organs. However, tumours are not typical endocrine organs (which can be modulated through negative feedback regulation), since the aberrant release of humoural mediators leads to paraneoplastic syndromes 74. This implies the continuous and unregulated production of hormones or peptides by tumours in patients with paraneoplastic syndromes.

#### Findings in late‐stage disease

Cancer cachexia is characterized by systemic inflammation, negative energy balance, involuntary loss of adipose tissue and skeletal muscle, and it is often associated with anorexia 80. Between 40 and 80% of cancer patients develop cachexia, particularly at advanced stages of the disease. Cancer anorexia‐cachexia syndrome (CACS) leading to progressive body weight loss arises from multiple interactions between tumours and the host response. The mechanisms underlying tumour–host interactions involve both humoural factors (*e.g*. TNF‐α, interleukin 1 [IL‐1] and IL‐6) and pro‐cachectic factors [*e.g*. proteolysis‐inducing factor (PIF) and lipid mobilization factor (LMF)] 81.

Tumour necrosis factor alpha, IL‐1 and IL‐6 can induce systemic inflammation and insulin resistance 71. During insulin resistance, TNF‐α increases gluconeogenesis, lipolysis and proteolysis, which result in decreased protein, lipid and glycogen synthesis 81. Interleukin‐1 and IL‐6 reduce insulin production and increase the levels of glucagon, cortisol and catecholamines in patients with CACS, leading to a hypercatabolic metabolism 71. In addition, the production of pro‐cachectic factors, including PIF and LMF, exerts direct catabolic effects on host tissues. Proteolysis‐inducing factor induces skeletal muscle breakdown *via* NF‐κB and the ubiquitin‐proteasome pathway 82. Lipid mobilization factor causes white adipose tissue wasting by sensitizing adipocytes to lipolytic stimuli 82.

Moreover, Cori cycle activity is increased in patients with malignancy 83, 84. Elevated glucose utilization with lactate production is core features of cancer cells 2. Actually, lactate levels in patients with malignancy are usually normal 84, since lots of lactate entering the blood to glucose in the liver 84, 85. This cyclic metabolic pathway, which links tumour glycolysis and host gluconeogenesis, is referred to as the ‘Cori cycle’ 86. Gluconeogenesis from lactate, which is an energy‐requiring process, contributes to excessive energy expenditure of the host 87.

Energy producing organs can detect the body's energy demand and subsequently generate signals to stimulate energy production, thereby providing energy to organs undergoing catabolic processes. During this process, dynamic energy homeostasis can be achieved through coordination of multiple organs. Similarly, solid tumours can be viewed as ‘organs’ 88. Solid tumours consist of cancer cells, stromal components, vasculature and immune cells. It is believed that interactions between the different components of cancer tissues are complex and that tumours can even interact with distal organs 88.

Some cachectic factors, such as cytokines, can inhibit the neuropeptide Y (NPY) pathway or imitate the negative feedback action of leptin on the hypothalamus, resulting in anorexia. With respect to energy production, anorexia is disadvantageous for cancer tissue, and mice bearing C26 tumours have been shown to increase their food intake following body weight loss 89. Moreover, gene expression during orexigenic differed from gene expression during anorexigenic growth. Tumour bearing (TB) mice showed increased expression of orexigenic NPY and agouti‐related protein 89 and decreased expression of anorexigenic pro‐opiomelanocortin and cholecystokinin 89. Furthermore, serotonin levels in the brain were lower in TB mice, but the levels of dopamine were unaffected 89. It is likely that the hypothalamic systems that regulate appetite in TB mice also control response and adaptation to changes in energy balance driven by tumour growth. Similarly, the neuroendocrine regulation of appetite by bidirectional signalling between the gut and brain is modified in rats by transplants of human hepatoblastoma and neuroblastoma 90. Thus, the level of ghrelin (an appetite stimulator) is elevated, while levels glucagon‐like peptide and peptide tyrosine‐tyrosine (appetite suppressors) are both reduced 90.

In addition to studies investigating CACS‐related factors, recent studies indicate that adipocytes provide fatty acids to ovarian cancer cells to fuel mitochondrial β‐oxidation 91, and omental adipocytes (through the mediation of adipokines such as IL‐8) stimulate homing, migration and invasion of ovarian cancer cells 92. These results indicate a long‐distance, reciprocal relationship between solid tumours and their host tissues.

Overall, cancer tissues behave like metabolic dictators, controlling energy homeostasis in order to satisfy their metabolic needs.

#### Basic research into mechanisms used by endocrine organ–like tumours

In addition to those observations described above, a number of basic research studies support our hypothesis of ‘endocrine organ–like tumour (EOLT)’ (Table 1).

**Table 1:** Summary of the evidence for our EOLT hypothesis

Early during cancer development	At a late stage in cancer development	Basic studies
Paraneoplastic syndromes 1. Secretion of hormones, peptides or cytokines 74 2. Cushing syndrome before cancer is diagnosed 75 3. In 85% of cases, the diagnosis of diabetes mellitus or hyperglycaemia precedes the diagnosis of pancreatic cancer by 2–3 years 78	Cancer cachexia 80 1. Systemic inflammation 2. Negative energy balance 3. Involuntary loss of adipose tissue and skeletal muscle Production of catabolic mediators such as proinflammatory cytokines (interleukins, interferon‐γ, tumour necrosis factor‐α, NF‐κB) 118, 119 Cancer cachexia and Cori cycle 83, 84, 86, 87 Increased food intake with body weight loss in mouse tumour model 89 Neuroendocrine regulation of appetite in human beings with hepatoblastoma and in neuroblastoma‐transplanted rats 90	27‐Hydroxycholesterol promotes breast cancer growth through oestrogen receptor‐dependent mechanisms and spurs its metastasis *via* liver X receptor 99 25‐Hydroxycholesterol inhibits IL‐1β, antagonizes sterol response element–binding protein and then represses IL‐1–activating inflammasomes 100 The gut microbiota promote hepatocellular carcinoma in the late stage through the lipopolysaccharides‐Toll‐like receptor 4 pathway 111 Intestinal microbiota, bile acids, nutrients (diets) and epithelial mucus can modulate immune responses, gut hormone synthesis and neuron activities to alter host metabolism and tumour energy expenditure 112 Microbial modification of bile acids can influence liver disease and result in metabolic syndrome *via* farnesoid X receptor and TGR5 signalling 116

### Tumour promoting lipids as regulators of the cancer macroenvironment

Recent studies indicate that lipid metabolites can act as metabolic messengers in interorgan crosstalk and modulate metabolic homeostasis 92. Similar to the way that hormones act on proximal organs, these molecules coordinate the regulation of energy homeostasis across tissues 92. For instance, Randle *et al*. found that metabolic signals from fat oxidation in the mitochondria inhibit glycolytic enzymes and thereby glucose utilization 93. Liu *et al*. discovered that hepatic *de novo* lipogenesis affects muscle fatty acid metabolism *via* the PPARδ pathway 94, 95. Furthermore, many lipids, including palmitoleate, eicosanoids and muscle‐derived β‐aminoisobutyric acid, target different tissues that mediate metabolic homeostasis, including adipocytes, immune cells and muscles 96, 97, 98.

Nelson *et al*. showed that 27‐hydroxycholesterol (27HC), a primary metabolite of cholesterol, promotes breast cancer cell growth through oestrogen receptor and breast cancer cell metastasis *via* the liver X receptor 99. In human breast cancer samples, the expression of CYP27A1, a cytochrome P450 oxidase required for the conversion of cholesterol to 27HC, correlates with tumour grade 93. In high‐grade tumours, both tumour cells and tumour‐associated macrophages highly express CYP27A1, creating an autocrine and paracrine milieu for production of 27HC, respectively 99. Recently, Reboldi *et al*. demonstrated that 25‐HC inhibits IL‐1β induction by type I interferon. 25‐HC antagonizes the processing of sterol response element–binding protein to suppress IL‐1β expression and to repress IL‐1–activating inflammasomes 100. As a participant in immune responses to viruses 100 and in the amplification of inflammation 101, 25‐HC production is activated by type I interferons and lipopolysaccharides (LPS), and 25‐HC is subsequently secreted during viral infection 102.

The novel linkage of cholesterol metabolism to diet, infection and cancer development provides support for our hypothesis. Further studies will determine if cholesterol production and metabolism through a tumour‐specific route is critical. Collectively, these studies suggest that bioactive lipid molecules might link cancer signalling to modulation of metabolic homeostasis during tumour development.

### Enteromicrobiota as a regulator of the cancer macroenvironment

Growing evidence suggests that along with excessive food intake and genetic polymorphisms, intestinal microbiota can contribute to obesity 103, 104, 105 and the development of diabetes, heart disease and cancers 106. Several studies show that translocation of intestinal bacteria can promote metabolic endotoxemia 107, which has been associated with insulin resistance 108. For example, Cox *et al*. found that altering the intestinal microbiota early in life by using low‐dose penicillin has metabolic consequences 109. Studies also show that the transfer of the gut microbiota from obese or lean donors influences the metabolic phenotype of the recipient 110. Moreover, Dapito *et al*. demonstrated the gut microbiota promote hepatocellular carcinoma (HCC) development in its late stages through the LPS‐Toll‐like receptor 4 (TLR4) pathway 111 but found no evidence that intestinal microbiota and TLR4 have an effect on the initiation of HCC 111. They also showed that resident liver cells, including hepatic stellate cells and hepatocytes, and non‐bone‐marrow‐derived cells, such as macrophages, mediate TLR4‐dependent tumour promotion in an NF‐κB dependent manner 111. In addition, host metabolism and energy balance can be regulated by the interplay between the intestinal microbiota, bile acids, nutrients and the epithelial mucus, which, in turn, modulate immune responses, gut hormone secretion and neuronal activity 112. For instance, diet‐derived fibres can be broken down and fermented to short‐chain fatty acids by gut bacteria 113. Short‐chain fatty acids not only can serve as an energy source for the epithelium and liver but also as mediators of the immune response 114. On the other hand, bile acids (which are made from cholesterol and secreted from the liver and gut as glycine, taurine or sulphate conjugates) are mostly reabsorbed in the ileum. But reabsorption is inhibited if the bile acids undergo deconjugation by intestinal microbiota 115. Bile acids can influence the absorption of fats and vitamins, and they can recognize the nuclear farnesoid X receptor (FXR) and the G‐protein–coupled receptor TGR5 116. These properties allow bile acids to inhibit bacterial proliferation directly and augment antimicrobial gene expression of the host cell indirectly 116.

Indeed, microbial modification of bile acids can influence liver disease and result in metabolic syndrome *via* FXR and TGR5 signalling 116. Research also suggests that most HCC results from chronic liver injury 117. Based on these studies, we suggest that the microbial–host relationship evolves over the course of tumour initiation and progression.

## Conclusions

**Figure 1:**
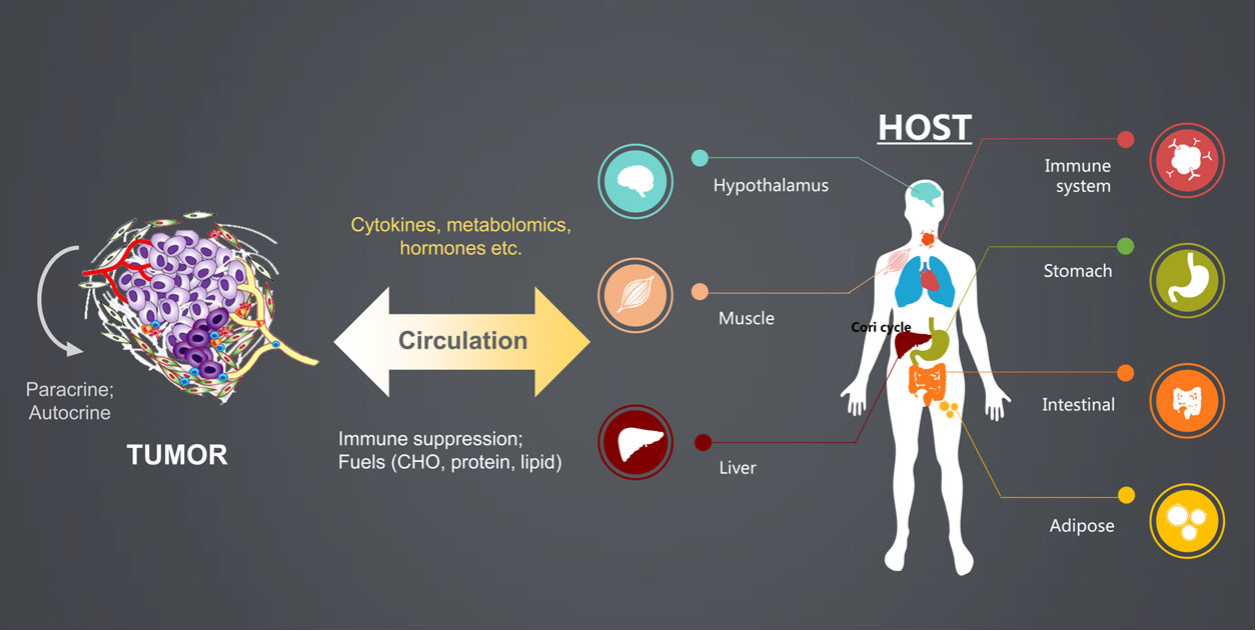
The schematic illustration of ‘EOLT hypothesis’. This article proposed a model of tumour–host interaction, by which tumour might disrupt systemic homeostasis and energy expenditure. Initially, the tumour is driven by oncogenic signals and the microenvironment trophic factors, *e.g*., paracrine or autocrine. In advance, the tumour anarchy could expand to release chemical factors, *e.g*., cytokine, metabolites and hormone into circulation. Those factors could further control homeostasis, including hypothalamus, muscle, immune system, liver and adipose tissues. For example, the appetite centre in CNS and hypothalamus could be altered throughout cancer development. And the systemic immune surveillance machinery might be suppressed by tumour. Lactate derived from glycolysis could be recycled to glucose *via* the Cori Cycle in the liver. The muscle and adipose tissue wasting could also occur in the cancer cachexia state under the challenge of tumour‐derived factors. On the contrary, the peripheral organs could also produce chemical signals to promote tumour. For example, the nutrient breakdown from liver, muscle and adipose could maintain a high level of fuel (carbohydrate, protein, lipid, … *etc*.) for feeding tumour. In addition, host metabolism and energy balance can be regulated by the interplay between the intestinal microbiota, bile acids, nutrients and the epithelial mucus, which, in turn, modulate immune responses, gut hormone secretion and neuronal activity. The peripheral immune function could be compromised to allow tumour progression. This tumour–host interaction is hypothesized, start in early stage, to be evolutional throughout cancer progression.

Most of cancer metabolism studies concentrate on the tumour itself or the interaction between tumour and its microenvironment. Obviously, the current concept of cancer metabolism does not account for the tremendous energetic demand of cancer nor explain systemic metabolism alteration. Therefore, we hypothesize that solid tumours can behave as metabolic dictators. One of best example in support our hypothesis is cancer cachexia. Cancer cachexia (tissue wasting) links regulation of the macroenvironment of tumours to the entire organism. A range of mediators produced by solid tumours influence metabolism of the host and result in significant and progressive energy loss from host tissue in the final stages of cancer. More importantly, an interaction of tumour glycolysis and host gluconeogenesis, called the ‘Cori cycle’, can explain energy transferred from distant organs to solid tumours and significant energy loss during cancer cachexia. Furthermore, patients with pancreatic cancer develop hyperglycaemia at an early stage. Studies suggest that pancreatic cancer cells secrete soluble factors that can impair β‐cell function and cause hyperglycaemia. Interestingly, pancreatic cancer resection ameliorates diabetes. Additionally, we provide some possible lipid metabolites that may play a role as a signalling molecule in interorgan crosstalk and regulate metabolic homeostasis. In this article, we hypothesized that regulation of the tumour macroenvironment continuously evolves over the entire course of tumour development. We reviewed the literature related to our hypothesis that solid tumours can behave as metabolic dictators (schematically illustrated in Fig. 1). Studies on paraneoplastic syndrome and cancer cachexia–anorexia syndrome showed that release of a number of cancer cell factors influence host metabolism. However, these studies do not show how solid tumours and host tissues interact to dictate metabolic homeostasis. It is particularly unclear whether the bidirectional interactions between tumours and surrounding tissues are regulated in a feedback manner. It is of great interest to determine how solid tumours regulate systemic homeostasis and vice versa. Additional studies to provide insights into the establishment of a tumour‐supportive macroenvironment during tumour development would benefit early cancer diagnosis, prevention and disease intervention.

## Funding

This work was supported by grants from the Taiwan Ministry of Sciences and Technology (MOST103‐2314‐B‐039‐034, 103‐2321‐B‐039‐004 and 104‐2628‐B‐039‐001‐MY4), Taiwan National Health Research Institution (NHRI‐EX104‐10214BC), China Medical University (CMU103‐BC‐5) and Taiwan Ministry of Health and Welfare Clinical Trial and Research Center of Excellence (MOHW104‐TDU‐B‐212‐113002).

## Conflicts of interest

The authors declare that they have no competing interests.

## Author contribution

YM Lee performed the literature search and wrote the article. WC Chang was involved in article discussion and editing. WL Ma initiated, supported the study, edited and gave final approval of the article.
